# The dataset on the characteristics of the intracerebral hemorrhage patients treated by endoscopic hematoma removal or craniotomy

**DOI:** 10.1016/j.dib.2020.106387

**Published:** 2020-10-08

**Authors:** Masahito Katsuki, Yukinari Kakizawa, Akihiro Nishikawa, Yasunaga Yamamoto, Toshiya Uchiyama

**Affiliations:** Department of Neurosurgery, Suwa Red Cross Hospital, 5-11-50, kogandori, Suwa, Nagano, Japan

**Keywords:** Intracerebral hemorrhage, Endoscopic hematoma removal, Craniotomy, Prognostic factor, Temporal muscle, Laboratory test, Stroke

## Abstract

These data present the characteristics of 148 intracerebral hemorrhage (ICH) patients surgically treated. We retrospectively collected data from the medical records of Suwa Red Cross Hospital, including neurological and physiological symptoms, laboratory data, radiological data on admission, complication rate, Glasgow Coma Scale scores on admission or postoperative day 7, and modified Rankin Scale scores at 6 months. Our two articles on the endoscopic hematoma removal and craniotomy for ICH were based on these data [1,2]. This dataset includes detailed laboratory data and radiological features, and it would be useful for reference value for other neurosurgeons or further analysis.

## Specifications Table

**Table:** 

Subject	Clinical Neurology
Specific subject area	Neurosurgery
Type of data	Table
How data were acquired	We investigated the medical records of Suwa Red Cross Hospital, and collected data.
Data format	Raw, and partially averaged.
Parameters for data collection	From the medical records, we collected objective data, such as laboratory data, physiological data, and neurological symptoms. Radiological information, such as temporal muscle thickness, area [3], [4], [5], [6], and hematoma volume [7] on the computed tomography (CT) image, could be less objective, so the averages of the two investigators were used.
Description of data collection	From the intracerebral hemorrhage (ICH) databases of Suwa Red Cross Hospital, we retrospectively retrieved the data from all 148 patients with ICH treated by endoscopic hematoma removal or craniotomy. We collected data regarding neurological and physiological symptoms and laboratory data on admission from the medical records. Radiological information was acquired using CT images. We also investigated the complication rate, the Glasgow Coma Scale on postoperative day 7, and the modified Rankin Scale at 6 months.
Data source location	Institution: Suwa Red Cross Hospital City/Town/Region: Suwa, Nagano Country: Japan Latitude and longitude (and GPS coordinates) for collected samples/data:] 36.0430059, 138.1068495
Data accessibility	With the article
Related research article	No. 1 Masahito Katsuki, Yukinari Kakizawa, Akihiro Nishikawa, Yasunaga Yamamoto, Toshiya Uchiyama Endoscopic hematoma removal of supratentorial intracerebral hemorrhage under local anesthesia reduces operative time compared to craniotomy [1] Sci Rep. 10 (2020) 10389. doi:https://doi.org/10.1038/s41598-020-67456-x. No. 2 Masahito Katsuki, Yukinari Kakizawa, Akihiro Nishikawa, Yasunaga Yamamoto, Toshiya Uchiyama Total Protein and Neuronavigation Are Novel Prognostic Factors of Endoscopic Hematoma Removal for Intracerebral Hemorrhage [2] J Stroke Cerebrovasc Dis. 29 (2020) 105050. doi:10.1016/j.jstrokecerebrovasdis.2020.105050.

## Value of the Data

• Data includes detailed characteristics of the ICH patients treated by endoscopic hematoma removal or craniotomy.
• This data can be used as a reference value for neurosurgeons when they treat their ICH patients.
• The data of temporal muscle thickness/area of the ICH patients can be used to compare healthy patients to investigate the meaning of temporal muscle, focusing on nutrition [8,9] and sarcopenia [10].

## 1 Data Description

The dataset in this table describes the characteristics of the ICH patients. The detailed data are available in the supplementary file.

**Table:** Characteristics of ICH patients who were surgically treated in Suwa Red Cross Hospital from 2012 to 2019.

Variables (*n* = 148)	Median (range)†
Sex, female: male	62:86
Age	74 (36–95)
Hematoma at the basal ganglia	79 (53%)
Hematoma at the subcortex (superficial lobar hemorrhage)	55 (37%)
Hematoma at the cerebellum	14 (10%)
Intraventricular hematoma	79 (53%)
GCS score on admission	10 (3–15)
Systolic blood pressure (*n* = 141) (mmHg)	166 (101–300)
History of smoking (*n* = 105)	43 (41%)
History of heavy drinking (*n* = 107)	23 (21%)
History of hypertension (*n* = 145)	123 (85%)
History of diabetes mellitus (*n* = 144)	25 (17%)
History of dyslipidemia (*n* = 144)	48 (33%)
Intake of antiplatelet drug (*n* = 147)	28 (19%)
Intake of anticoagulant drug	25 (17%)
Endoscopy: Craniotomy	75:73
Operative time (min)	77 (30–272)
GCS score on postoperative day 7 (*n* = 140, others died)	13 (3–15)
Modified Rankin Scale at 6 months	4 (1–6)
Death during hospitalization	26 (18%)
Rebleeding	11 (7%)
Other complications except for rebleeding (infectious diseases, convulsion, heart failure, cerebral infarction, hydrocephalus)	52 (35%)
Temporal muscle thickness (mm)	5.5 (1.0–11.5)
Temporal muscle area (mm^2^)	278.5 (10.0–1042.5)
Apparent destruction of the pyramidal tract	96 (65%)
Preoperative hematoma volume (mL)	102 (10–397)
Postoperative hematoma volume (mL)	2 (0–350)
Removal rate	98% (0–100%)
Total protein (mg/dL) (*n* = 142)	7.1 (5.4–9.5)
Blood glucose (mg/dL) (*n* = 127)	144 (86–397)

† The Shapiro-Wisk test revealed all continuous variables were not with a normal distribution. The detailed data are available in the supplementary file.

## 2 Experimental Design, Materials and Methods

We investigated our medical record and retrospectively retrieved the data of 148 ICH patients surgically treated from 2009 to 2019. All patients had been independent in activities in daily living before the onset of ICH. The diagnosis of ICH was based on the clinical history and the presence of ICH on CT. The inclusion criteria for the study were; 1) ICH patients in the basal ganglia, subcortex (superficial lobe), or cerebellum; 2) patients indicated for surgery according to the Japanese Guidelines for the Management of Stroke 2009 [11] and 2015 [12]; and 3) interval between onset and hematoma removal <24 h. The exclusion criteria were; 1) ICH due to tumor, trauma, aneurysm, arteriovenous malformation, and hemorrhage after infarction; and 2) patients who had thalamic or caudate head hemorrhage with intraventricular hemorrhage treated by flexible neuroendoscope for removing intraventricular hematoma only.

All patients received standard management according to the Japanese Guidelines for the Management of Stroke 2009 [11] and 2015 [12]. First, they were treated with nicardipine to control blood pressure under 140 mmHg. In patients under anticoagulation therapy, prothrombin time was normalized by vitamin K and/or fresh frozen plasma. The criteria for surgical indication were as follows: (1) patients with hematoma (>30 mL) in the basal ganglia who were deteriorating neurologically; (2) patients with superficial lobar hemorrhage within 1 cm of the cortical surface and with disturbance of consciousness; and (3) patients with cerebellar hemorrhage >3 cm in diameter who were deteriorating neurologically or who had brainstem compression and/or hydrocephalus from ventricular obstruction. Rehabilitation and nutritional support were started as soon as possible after surgery, and complications were prevented or treated. Antithrombotic drugs were discontinued postoperatively for several days, depending on the patient's condition and comorbidities.

We collected data about neurological and physiological symptoms, clinical course, and laboratory data on admission. We measured the hematoma volume and the removal rate of the hematoma from the head CT at admission and immediately postoperatively. The hematoma volume was calculated using ABC/2 method [7]. We also investigated the presence of obvious destruction of the pyramidal tract on CT. We checked the primary motor area, corona radiata, posterior limb of the internal capsule, and cerebral peduncle. When one of them was obviously destroyed by the hematoma as a high-density area, we defined obvious destruction of the pyramidal tract; when destruction was equivocal, we defined that the pyramidal tract was not destroyed.

Glasgow Coma Scale scores on day 7, modified Rankin Scale scores at 6 months postoperatively, and postoperative complications were investigated by the medical records, telephonic, or personal interview.

## Ethics Statement

We obtained informed consent for the study and publishment from all patients or their families. The hospital ethics committee approved this study.

## Declaration of Competing Interest

The authors declare that they have no known competing for financial interests or personal relationships which have, or could be perceived to have, influenced the work reported in this article.

## Appendix Supplementary materials

Image, application 1

## Footnotes

Supplementary material associated with this article can be found in the online version at doi:10.1016/j.dib.2020.106387.
